# Portrait of WWP1: the current state in human cancer

**DOI:** 10.3389/fcell.2024.1516613

**Published:** 2025-01-30

**Authors:** Jiaming Lei, Jun Chen, Wenwen Yu, Qing Wu, Shuang Jing, Yuanguang Tang, Li Lin, Meichun Hu

**Affiliations:** ^1^ Key Laboratory of Environmental Related Diseases and One Health, School of Basic Medical Sciences, Xianning Medical College, Hubei University of Science and Technology, Xianning, Hubei, China; ^2^ The Central Hospital of Ezhou, Affiliated Hospital of Hubei University of Science and Technology, Ezhou, Hubei, China; ^3^ State Key Laboratory of Genetic Resources and Evolution, Key Laboratory of Healthy Aging Research of Yunnan Province, Kunming Key Laboratory of Healthy Aging Study, KIZ/CUHK Joint Laboratory of Bioresources and Molecular Research in Common Diseases, Kunming Institute of Zoology, Chinese Academy of Sciences, Kunming, Yunnan, China

**Keywords:** WWP1, human cancer, upstream factor, downstream substrate, targeted therapy

## Abstract

WWP1, a member of the C2-WW-HECT E3 ligase family, is an E3 ubiquitin-protein ligase containing WW domains. This enzyme plays a critical role in regulating diverse cellular processes. Its expression is modulated by various factors and non-coding RNAs, resulting in ubiquitination that affects substrate protein degradation. WWP1 demonstrates a dual function, acting predominantly as an oncogene in tumors but occasionally as a tumor suppressor. This review summarizes WWP1’s biological roles, therapeutic potential in oncology, upstream regulatory factors, and downstream substrates. It aims to promote research on WWP1’s antitumor effects, improve understanding of its role in tumorigenesis, and support the development of targeted therapies.

## 1 Introduction

Ubiquitin, a 76-amino acid protein, categorizes intracellular proteins and selects targets for specific modifications through ubiquitination. As a critical post-translational modification, ubiquitination maintains cellular protein homeostasis (Senft et al., 2018; Sampson et al., 2023). Dysregulated ubiquitination is associated with DNA damage repair, cell cycle regulation, gene expression, and signal transduction (Dagar et al., 2023). This multi-step enzymatic process involves ubiquitin-activating enzymes (E1s), ubiquitin-conjugating enzymes (E2s), and ubiquitin ligases (E3s), facilitating protein-protein interactions and cell signaling depending on the type of ubiquitin linkage (Hershko and Ciechanover, 1998; Cappadocia and Lima, 2018). The human genome encodes two E1 enzymes, approximately 40 E2 enzymes, and over 600 E3 enzymes. E3 ubiquitin ligases determine the specificity and modification sites of target proteins, catalyzing the transfer of ubiquitin from E2 enzymes to substrate lysines, forming covalent bonds (Berndsen and Wolberger, 2014; Stewart et al., 2016).

E3 ubiquitin ligases are classified into RING-type and HECT-type ligases. RING-type E3 ligases lack intrinsic ligase activity (Sluimer and Distel, 2018; Asano et al., 2023), whereas HECT-type ligases possess this activity. HECT-type ligases are further divided into three families based on their N-terminal domains: the NEDD4 family (9 members), the HERC family (6 members), and other E3 ligases (13 members) (Bernassola et al., 2008; Bernassola et al., 2019; Singh et al., 2021). The NEDD4 family contains 2 to 4 WW domains, which are structural and signaling modules crucial for RNA transcription, protein transport, receptor signaling, and cytoskeletal regulation. These domains specifically recognize and bind proline-rich motifs (PRMs) and phosphorylated serine/threonine-proline sites, facilitating protein-protein interactions (Singh et al., 2021; Tian et al., 2023; Rotin and Prag, 2024).

WWP1, a member of the NEDD4 family, regulates various cellular processes. Studies have shown that WWP1 is frequently overexpressed or amplified in numerous cancers, promoting tumor growth, cancer cell proliferation, migration, and cell cycle progression while inhibiting apoptosis (Zhi and Chen, 2012). WWP1’s critical role in tumor regulation lies in its interactions with ubiquitination substrates. This review explores WWP1’s upstream regulatory molecules, downstream substrates, and associated signaling pathways in human cancers (Llovet et al., 2018). Additionally, it provides an updated analysis of recent research on WWP1’s role in various cancers, highlighting its therapeutic potential in oncology.

## 2 WWP family in cancer

The WW family of proteins, including WWP1, WWP2, and WWP3, plays a critical role in regulating cellular processes through WW domains that mediate interactions with specific protein motifs (Li Y et al., 2024). While WWP1 has been extensively studied for its role in cancer via ubiquitin-mediated degradation, WWP2 shares structural and functional similarities with WWP1 but performs distinct roles, particularly in regulating the cell cycle and protein degradation (Lee and Liou, 2018; Liu et al., 2020). WWP2 has been implicated in several cancers, influencing tumor progression by modulating key cell cycle proteins and apoptotic pathways (Lee et al., 2019). In contrast, WWP3, though less studied, plays an important role in the nervous system, particularly in neuronal development and function. Despite their similarities, WWP1, WWP2, and WWP3 exhibit distinct expression patterns and functions across various tissues and pathological states (Das et al., 2015; Faber et al., 1998). Understanding their interactions and functional divergence is crucial to uncovering their potential as therapeutic targets in cancer and other diseases (Li Y. et al., 2024). Future research should explore how these proteins collaborate within cellular pathways and their potential for targeted cancer therapies (Hu et al., 2004).

Recent studies have highlighted the involvement of WWP family members, particularly WWP1, in regulating immune responses and critical cellular processes such as protein degradation, cell cycle progression, and apoptosis (Chen et al., 2020). As E3 ubiquitin ligases, WWPs play pivotal roles in modulating immune responses, including T-cell activation, cytokine production, and immune cell differentiation, which are essential for cancer progression and immune evasion. Furthermore, WWP family members have been implicated in regulating the tumor microenvironment, influencing both tumor and immune cells (Chen and Matesic, 2007; André and Springael, 1994). These functions suggest that WWPs play a central role in cancer biology beyond their well-established roles in protein degradation and cellular regulation.

### 2.1 Mutation status and survival in different cancers

Mutations in WWP family genes, including WWP1, have been observed in various cancers and are often associated with poor clinical outcomes (André and Springael, 1994). For instance, in breast cancer, high WWP1 expression correlates with shorter overall survival (OS) and disease-free survival (DFS). A study by [Author et al., Year] demonstrated that patients with high WWP1 expression had significantly lower OS compared to those with low expression, with a hazard ratio (HR) of 1.6 (p < 0.05). Similarly, in colorectal cancer, WWP1 overexpression correlates with advanced tumor stages and poor prognosis, with survival analyses indicating significantly reduced survival rates in patients with high WWP1 levels.

In non-small cell lung cancer (NSCLC), the WWP1 R503W mutation has been linked to increased cancer cell proliferation and invasion (Wu et al., 2019; Xie et al., 2015; Yao et al., 2018). Survival analysis showed that high WWP1 expression was associated with worse prognosis and decreased 5-year survival rates, particularly in stage III and IV patients (Faber et al., 1998).

Moreover, in hepatocellular carcinoma (HCC), WWP2, another WWP family member, is frequently overexpressed, with higher levels correlating with tumor recurrence and lower survival rates. In ovarian cancer, WWP2 mutations significantly affect survival, with patients harboring WWP2 mutations exhibiting a median survival time of 12 months compared to 24 months in those without the mutation (Li Z et al., 2024).

In gastric cancer, elevated expression levels of both WWP1 and WWP2 are significantly associated with worse OS, particularly in early-stage tumors, suggesting a role for these proteins in the early metastatic spread of gastric cancer (Sluysmans et al., 2021).

In summary, the mutation status and RNA expression levels of WWP family members, particularly WWP1 and WWP2, are emerging as important prognostic factors in various cancers. High expression levels of WWP1 and WWP2 frequently correlate with poor prognosis, increased tumor invasiveness, and reduced survival rates in cancers such as breast, colorectal, lung, liver, and ovarian cancers. These findings underscore the potential of WWPs as biomarkers and therapeutic targets in cancer treatment.

## 3 Historical review of WWP1

**FIGURE 1:**
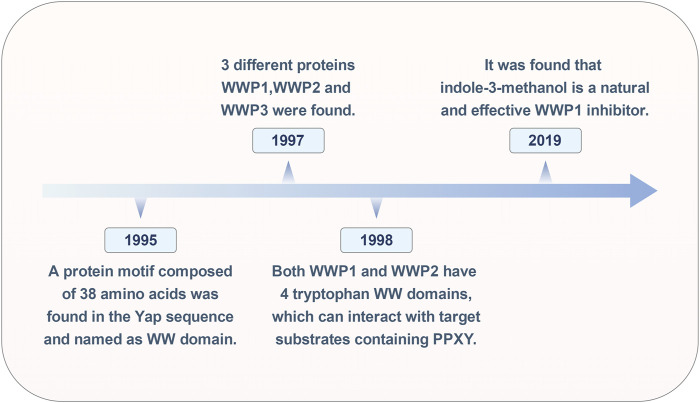
Historical timeline of the WWP1 milestone event.

The research on WWP1 (Figure 1) spans nearly 30 years. In 1995, Sudol and colleagues identified a 38-amino-acid protein motif within the yes-associated protein (Yap) sequence, characterized by four highly conserved aromatic amino acids, including two tryptophan residues, termed the WW domain (Chen and Sudol, 1995). In 1997, Pirozzi’s team used cloning of ligand targets (COLT) to screen cDNA expression libraries from human brain and bone marrow, identifying 13 positive clones encoding three distinct proteins: WWP1, WWP2, and WWP3 (Pirozzi et al., 1997). By 1998, Wood et al. reported that all five atrophin-1 interacting proteins (AIPs) contained WW domains that interact with the PY motif (PPXY) of AIP-1. Both WWP1 and WWP2 were found to possess four WW domains, enabling them to interact with substrates containing the PY motif (Wood et al., 1998).

In cancer treatment, while inhibiting oncoproteins remains a primary therapeutic strategy, identifying tumor suppressors for clinical application has proven challenging. In 2019, Lee and colleagues discovered that Indole-3-carbinol, a compound found in cruciferous vegetables, binds effectively to the HECT domain of NEDD4-1, with an equilibrium dissociation constant of approximately 88.1 μM, significantly inhibiting NEDD4-1 activity. Due to the structural similarity between the HECT catalytic domains of WWP1 and NEDD4-1, further testing revealed that Indole-3-carbinol serves as a natural inhibitor of WWP1 (Lee et al., 2019). Over time, WWP1 has been linked to various human diseases, particularly tumorigenesis. However, the full scope of WWP1’s functions remains unclear, necessitating further research.

## 4 Structure of WWP1

**FIGURE 2:**
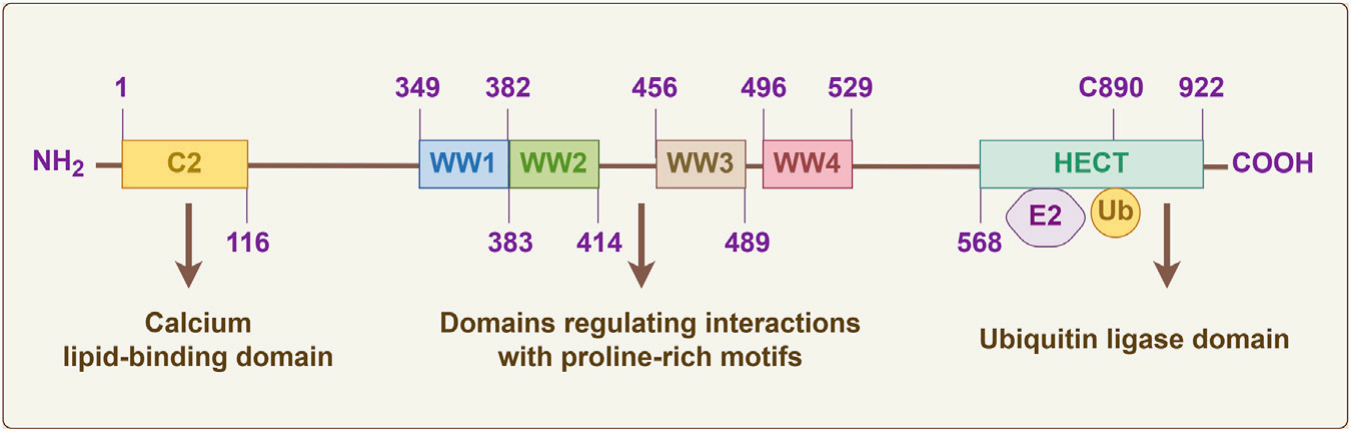
The diagram of WWP1 protein domains. The WWP1 protein, consisting of 922 amino acid residues, includes: an N-terminal C2 domain responsible for membrane and protein binding through its calcium lipid-binding domain; four WW domains (WW1, WW2, WW3, and WW4) in the central region, which facilitate interaction with substrate proline-rich motif proteins; and a C-terminal HECT domain responsible for ubiquitin-protein ligase activity. The catalytic activation site is located at cysteine-890.

The WWP1 protein, approximately 110 kDa in molecular weight, consists of 922 amino acid residues (Figure 2). As a key component of the ubiquitin ligase complex, WWP1 exhibits a distinct structure with an N-terminal C2 domain, four tandem WW domains, and a C-terminal HECT domain. The N-terminal C2 domain likely mediates protein-protein interactions (Wang et al., 2010). The four WW domains facilitate interactions with substrates containing PY motifs, which are marked with ubiquitin. WWP1 can also bind substrates lacking the PY motif, often requiring the ubiquitination of an adapter protein to bridge the interaction (Shah and Kumar 2021).

As shown in Figure 2, the C-terminal HECT domain interacts with E2 enzymes and is essential for E3 ligase activity. Unlike RING finger E3 ligases, HECT E3 ligases contain a catalytic cysteine (C) that forms a covalent isopeptide bond with ubiquitin. Specifically, the catalytic residue C890 in human WWP1 is crucial for transferring ubiquitin from WWP1 to the substrate protein (Verdecia et al., 2003; Zhi and Chen, 2012). This unique combination of domains endows WWP1 with diverse functional capabilities in cellular ubiquitination processes.

## 5 Functions of WWP1

**FIGURE 3:**
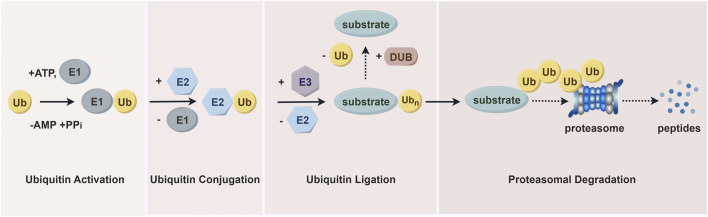
The ubiquitin-proteasome pathway. Proteins are first tagged with ubiquitin and subsequently recognized and degraded by the proteasome. This system includes ubiquitin, ubiquitin-activating enzymes (E1s), ubiquitin-conjugating enzymes (E2s), and ubiquitin-protein ligases (E3s), which mediate the ubiquitination process. The process also involves deubiquitinating enzymes (DUBs) and proteasomal degradation facilitated by the 26S proteasome.

The ubiquitin-proteasome system (UPS) is essential for degrading most intracellular proteins (Figure 3). This pathway involves two sequential steps. First, utilizing ATP as an energy source, ubiquitin is activated and linked to E1 via a thioester bond, a process known as ubiquitin activation. In the second step, the ubiquitin-activating enzyme E1 transfers the activated ubiquitin to ubiquitin-conjugating enzyme E2. The final step is proteasomal degradation, where the 26S proteasome complex recognizes lysine 48- or 11-linked polyubiquitin chains and degrades ubiquitin-tagged substrates into small peptides (approximately 3–15 amino acids). These peptides are subsequently broken down into individual amino acids by cytoplasmic proteases, allowing the recycled ubiquitin to be reused (Fukasawa et al., 2012; Spano and Catara, 2023). As an E3 ubiquitin ligase, WWP1 plays a critical role in the UPS by ensuring the high specificity and precise regulation of ubiquitin-mediated substrate protein degradation.

**FIGURE 4:**
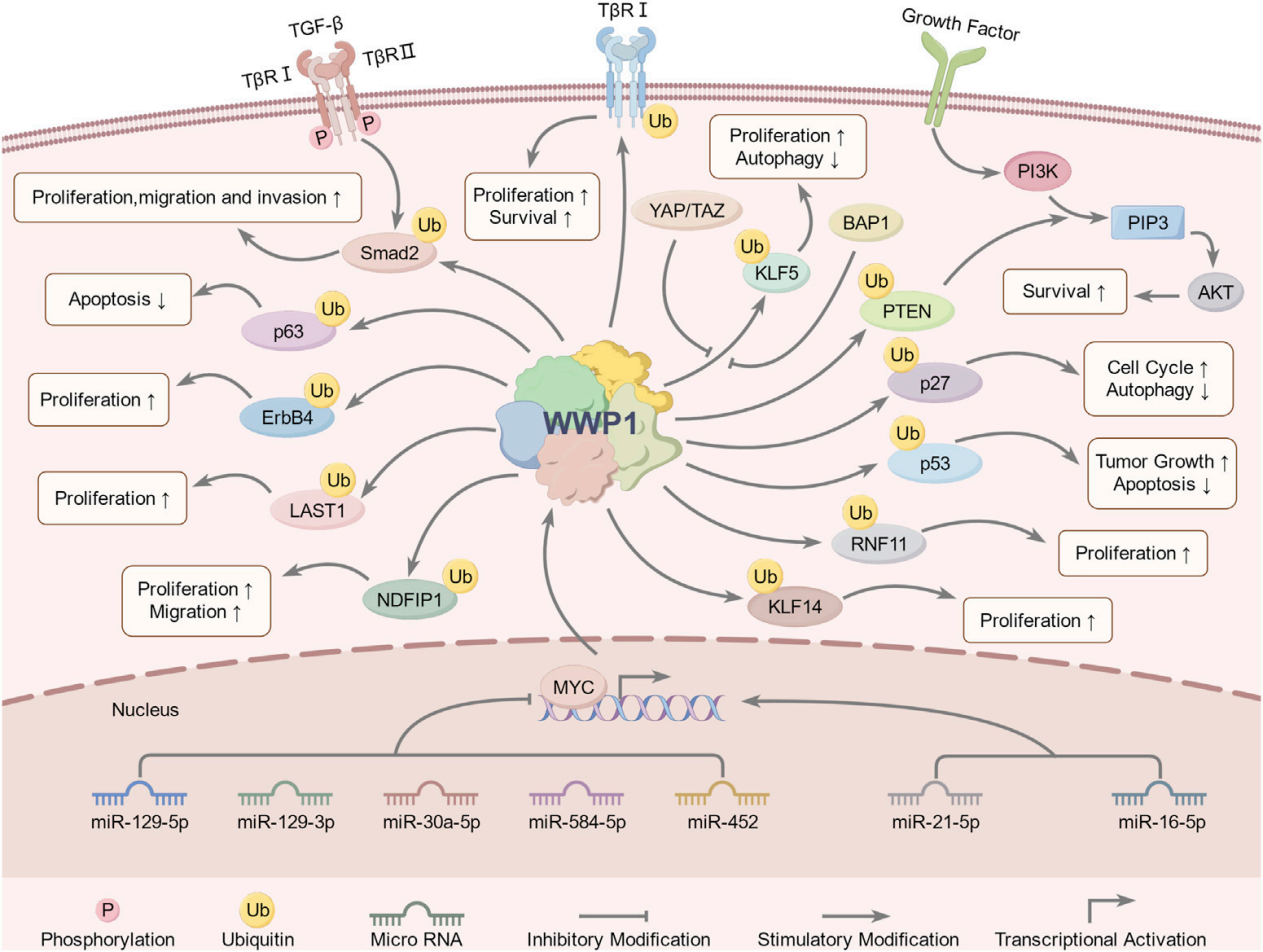
WWP1-involved signaling pathways and regulatory mechanisms. Upstream regulators of WWP1 include miR-30a-5p, miR-21-5p, miR-16-5p, miR-129-5p, miR-129-3p, miR-584-5p, miR-452, YAP, TAZ, MYC, and BAP1. WWP1 regulates downstream substrates through ubiquitination, including Smad2, PTEN, ErbB4, KLF5, LATS1, NDFIP1, p63, RNF11, TβRI, and p27. These pathways collectively regulate growth, proliferation, migration, invasion, apoptosis, autophagy, tumorigenesis, and other biological processes.

In disease contexts, WWP1 expression has significant implications (Figure 4). Its overexpression in cardiomyocytes has been linked to increased apoptosis, larger infarct sizes, and impaired cardiac function. In contrast, WWP1 inhibition reduces myocardial ischemic injury following ischemic myocardial infarction (MI). KLF15, a zinc finger transcription factor, regulates cardiomyocyte survival and function. WWP1-mediated degradation of KLF15 has been shown to increase p65 acetylation and activate MAPK inflammatory signaling in ischemic myocardium and hypoxia-treated cardiomyocytes (Shao et al., 2018; Lu et al., 2023). Additionally, WWP1 stabilizes disheveled segment polarity protein 2 (DVL2) through K27-linked polyubiquitination, a process critical for cardiac hypertrophy. WWP1 promotes K27-linked ubiquitin multichain assembly on DVL2, exacerbating cardiac hypertrophy via the DVL2/CaMKII/HDAC4/MEF2C pathway (Zhao et al., 2021).

Reports have highlighted the role of E3 ubiquitin ligase-mediated protein degradation in promoting the proteasomal breakdown of key positive regulators of osteoblasts. Enzymes such as Smurf1, Itch, and WWP1 facilitate the degradation of proteins like Runx2, JunB, and CXCR4, inhibiting their functions (Sudol et al., 1995). Consequently, WWP1 may act as a negative regulator of osteoblast activity by reducing the protein levels of osteoblast-positive regulators. This suggests that WWP1 inhibitors could represent a novel class of bone anabolic drugs for treating osteoporosis (Shu et al., 2013).

Recent studies have also shown elevated WWP1 levels in the white adipose tissue (WAT) of obese mouse models. Notably, WWP1 knockout mice exhibited improved systemic glucose metabolism and increased p-AKT levels in the liver, though levels in WAT and skeletal muscle remained unchanged. Liver weight and triglyceride content were also reduced in obese WWP1 knockout mice (Hoshino et al., 2020; Nozaki et al., 2023).

Beyond its role in human pathology, WWP1 has been shown to bind to Ptch1 and ubiquitinate Smo, influencing cilia dynamics in vertebrates (Lv et al., 2021; Mund and Pelham, 2010). These findings underscore the significance of WWP1 in various biological processes and highlight its potential as a focus of further research into its diverse functions.

## 6 Regulation of WWP1

WWP1 is a critical E3 ligase enzyme involved in ubiquitination, marking proteins for degradation or modification. Regulation of WWP1 expression significantly influences various cellular processes, particularly those related to cancer. Its regulation involves numerous upstream factors, making it vital to investigate these mechanisms to understand its role in cancer and other diseases. Non-coding RNAs, transcriptional regulators, and other factors impact WWP1 expression, thereby affecting diverse cancer types (Table 1).

**TABLE 1:** Upstream regulators of WWP1 and their effects in cancer.

Mechanism	Cancer	Upstream regulators	Function	References
Regulation of WWP1 by non-coding RNAs	Glioma	miR-30a-5p	Promotes the proliferation, migration and invasion of glioma cells.	Zhao (2019)
	IBD-related colorectal cancer	miR-21-5p miR-16-5p	Promotes the occurrence and progression of IBD-related colorectal cancer.	Zhou (2021)
	Gastric cancer	miR-129-5p miR-129-3p	Inhibit proliferation and migration *in vitro*.	Ma et al. (2019)
		miR-584-5p	Inhibits proliferation and promotes apoptosis, promotes cell senescence.	Li et al. (2017)
	Prostate cancer	miR-452	Inhibits the migration and invasion of prostate cancer cells.	Goto et al. (2016)
Transcriptional regulation of WWP1	Intrahepatic cholangiocarcinoma	MYC	Promotes cell growth and survival.	Li et al. (2022)
Other mechanism	Breast cancer	TAZ	Promotes the proliferation of breast cancer cells.	Zhao et al. (2012)
		YAP	Promotes the growth and survival of breast cancer cells.	Zhi et al. (2014)
	Melanoma	BAP1	Inhibits autophagy and promotes melanoma proliferation.	Jia et al. (2021)

### 6.1 Regulation of WWP1 by non-coding RNAs

Recent studies have identified upstream signaling pathways that regulate WWP1 expression (Figure 4). Various non-coding RNAs either upregulate or downregulate WWP1. For instance, in glioma tissues, decreased WWP1 levels correlated with increased miR-30a-5p and p65 phosphorylation levels, suggesting a negative correlation between WWP1 mRNA and miR-30a-5p expression. Notably, miR-30a-5p promoted glioma cell proliferation, migration, and invasion by targeting WWP1 (Zhao et al., 2019).

In patients with inflammatory bowel disease (IBD), increased levels of miR-16-5p and miR-21-5p (elevated in ulcerative colitis) suggested WWP1 as a target for these microRNAs, potentially contributing to IBD-associated colorectal cancer (CRC) (Zhou et al., 2021). In gastric cancer (GC), downregulated miR-129-5p and miR-129-3p were associated with WWP1 expression. Functional studies indicated that miR-129-5p binds to the coding sequence of WWP1 mRNA, inhibiting GC cell proliferation and migration *in vitro* and slowing tumor growth *in vivo* (Ma et al., 2019). Similarly, miR-584-5p inhibited GC cell proliferation and induced apoptosis by down-regulating WWP1 and activating the TGF-β signaling pathway (Li et al., 2017).

In prostate cancer (PCa), WWP1 is a direct target of miR-452, which is downregulated in PCa patients. Increased WWP1 expression negatively correlates with miR-452 levels, and miR-452 inhibits PCa cell proliferation and invasion by regulating WWP1 (Goto et al., 2016) (Table 1). These findings provide insights into the molecular mechanisms of cancer development and suggest potential therapeutic targets. Further research is required to explore the roles of WWP1 and its regulatory microRNAs in cancer pathogenesis and treatment.

### 6.2 Transcriptional regulation of WWP1

The MYC gene is one of the most frequently dysregulated oncogenes implicated in the initiation, progression, and advancement of various cancers (Duffy et al., 2021). Research by Lee et al. demonstrated that MYC is enriched in the promoter region of WWP1, promoting its expression and identifying WWP1 as a potential MYC target gene. Additionally, phosphatase and tensin homolog (PTEN), a commonly mutated, deleted, or silenced tumor suppressor in numerous cancers, has been linked to the WWP1-PTEN axis in MYC-induced tumorigenesis and progression (Li et al., 1997; Bonneau and Longy, 2000; Lee et al., 2019).

Further studies showed that WWP1 is highly expressed in intrahepatic cholangiocarcinoma (ICC), with elevated levels correlating with poor prognosis. WWP1 targets NEDD4 family interacting protein 1 (NDFIP1) for ubiquitination, promoting ICC cell proliferation, migration, and invasion. Importantly, MYC drives WWP1 upregulation in ICC, enhancing WWP1 expression to support ICC cell growth and survival through NDFIP1 ubiquitination (Li et al., 2022) (Table 1). These findings deepen our understanding of the interactions among MYC, WWP1, and PTEN in cancer development, highlighting promising therapeutic targets for future cancer treatments.

### 6.3 Other mechanisms involved in the regulation of WWP1

The Hippo signaling cascade includes two key kinases, MST1/2 and LATS1/2, along with upstream components such as Merlin/NF2, FRMD, and KIBRA, which are crucial for pathway regulation (Yin et al., 2013; Xu et al., 2016; Swaroop et al., 2021). MST1/2, supported by SAV1, activates LATS kinases by phosphorylating LATS1/2 and its cofactor MOB1. Activated LATS1/2 then phosphorylates YAP and TAZ, resulting in their inhibition via cytoplasmic translocation or degradation. Specifically, TAZ phosphorylation promotes β-TrCP-mediated ubiquitination and subsequent proteasomal degradation.

TAZ, a transcriptional coactivator, is negatively regulated by the Hippo pathway (Wang et al., 2009). It is frequently overexpressed in breast cancer (BC), driving cell proliferation, migration, and invasion (Chan et al., 2008). WWP1 recognizes and interacts with KLF5, a transcription factor highly expressed in BC, via its WW domain, leading to KLF5’s ubiquitination and proteasomal degradation (Chen et al., 2005). Interestingly, TAZ disrupts this interaction, enhancing BC cell proliferation by preventing WWP1-mediated KLF5 degradation.

YAP, another coactivator and primary effector of the Hippo pathway, is overexpressed in various tumors, including BC (Tong et al., 2006). BRCA-associated protein-1 (BAP1), a deubiquitinating enzyme and tumor suppressor, regulates processes such as DNA repair and cell cycle control (Louie and Kurzrock, 2020). Studies have shown that WWP1 is underexpressed in melanoma, where KLF5 levels are elevated. BAP1 counteracts WWP1-mediated KLF5 ubiquitination, promoting melanoma development by stabilizing KLF5 within the BAP1/HCF-1 complex (Misaghi et al., 2009; Jia et al., 2021) (Table 1). This complex contributes to cell cycle progression by inhibiting p27 expression, thereby driving oncogenic effects in BC (Qin et al., 2015).

## 7 Downstream substrates of WWP1

Extensive evidence suggests that WWP1 mediates the ubiquitination of protein substrates, including Smad2, KLF5, p63, ErbB4, and RNF11 (Table 2) (Figure 4). Many WWP1 substrates contain PY motifs, such as ErbB4, KLF5, and LATS1. However, WWP1 also indirectly affects substrates lacking PY motifs by utilizing adapter proteins like KLF14 and p27. Additionally, WWP1 applies monoubiquitin or diverse polyubiquitin chains to numerous substrates. Notably, K48-linked polyubiquitin chains serve as recognized degradation signals by the 26S proteasome, indicating that most degradation-related modifications involve K48-linked polyubiquitination (Grice and Nathan, 2016; Kuang et al., 2021). Despite extensive studies, the exact inhibitory mechanisms of WWP1 in tumorigenesis and its specific binding patterns with substrates remain unclear. This review aims to classify WWP1’s ubiquitinated substrates, establishing a foundation for future research in cancer treatment and diagnostics.

**TABLE 2:** WWP1 modulates substrates and its effects in cancer.

Mechanism	Cancer	Substrates	Function	References
PY Motif containing substrates	Breast cancer	ErbB4	Promotes the proliferation and survival of breast cancer cells.	Li et al. (2009)
		KLF5	Inhibits of proliferation and survival of breast cancer cells.	Zhao et al. (2012); Zhi et al. (2012)
		LATS1	Promotes the proliferation of breast cancer cells.	Yeung et al. (2013)
	Prostate cancer	Smad2	Inhibits the TGF-β pathway and promotes the proliferation of prostate cells.	Chen et al. (2007)
	Melanoma	KLF5	Inhibits the proliferation, migration and invasion of melanoma and promotes its autophagy.	Jia et al. (2021)
	Intrahepatic cholangiocarcinoma	NDFIP1	Promotes the proliferation and migration of intrahepatic cholangiocarcinoma.	Li et al. (2022)
		p63	Inhibits cell apoptosis.	Li et al. (2008)
		RNF11	Promotes cell proliferation.	Chen et al. (2008)
Non-PY motif containing substrates	Prostate cancer	TβRI	Inhibits the TGF-β pathway and promotes the proliferation of prostate cells.	Massagué and Like (1985)
		PTEN	Promotes the survival of prostate cancer cells.	Lee et al. (2019)
	Breast cancer	PTEN	Promotes the growth of breast cancer cells.	Kishikawa et al. (2021)
	Acute myeloid leukemia	p27	Promotes cell cycle, inhibits autophagy.	Sanarico et al. (2018)
	Liver cancer	KLF14	Promotes the proliferation and migration of liver cancer.	Zhang et al. (2024)
	Hepatocellular carcinoma	p53	Promotes cell growth and inhibits apoptosis.	Cheng et al. (2014)

### 7.1 Substrates containing PY motif

The epidermal growth factor receptor (EGFR) family, including ErbB4, plays critical roles in various cancers, notably breast cancer (BC) (Roskoski, 2014). ErbB4, a 180 kDa glycoprotein, features a ligand-binding extracellular region, a hydrophobic transmembrane domain, and a cytoplasmic region containing a tyrosine kinase domain (Omerovic et al., 2004). Research has shown that WWP1 specifically interacts with the ErbB4-CyT1 subtype, targeting it for ubiquitination-mediated degradation. This interaction involves WWP1’s first and third WW domains and ErbB4’s second PY motif. Notably, ErbB4 activates the tumor suppressor BRCA1 and reduces the proliferation and survival of breast epithelial cells (Muraoka-Cook et al., 2006). WWP1 reduces endogenous ErbB4 levels in BC cell lines, promoting cell proliferation and survival.

KLF5, a key member of the KLF family, is also targeted by WWP1, which interacts with its WW domain, leading to KLF5 degradation and suppressed BC cell proliferation (Li et al., 2009). WWP1 acts as a negative regulator of LATS1, a serine/threonine kinase and tumor suppressor. By binding to WWP1’s WW domains, LATS1 undergoes polyubiquitination and degradation via the 26S proteasome pathway (Visser and Yang, 2010), reducing LATS1 levels and enhancing BC cell proliferation (Yeung et al., 2013). These findings underscore WWP1’s multifaceted role in modulating key proteins involved in cancer progression.

TGF-β, a multifunctional cytokine, regulates cell proliferation, differentiation, survival, and apoptosis through various intracellular pathways (Kaminska et al., 2005). Smad transcription factors are central to TGF-β signaling and cancer progression (Massagué et al., 2005). WWP1 negatively regulates this pathway by ubiquitinating and degrading critical components like Smad2 and TβRI, which in prostate cancer (PCa) results in WWP1 overexpression that inhibits TGF-β-induced gene expression and promotes cell proliferation (Chen et al., 2007).

NDFIP1, an adaptor for NEDD4 family proteins, interacts with WWP1 and has potential anti-tumor effects. However, WWP1-mediated ubiquitination reduces NDFIP1 levels, promoting intrahepatic cholangiocarcinoma (ICC) cell proliferation and migration (Li et al., 2022). Similarly, RNF11, overexpressed in various tumors (Li et al., 2008; Wojciechowski et al., 2018), interacts with WWP1’s WW domains via its PY motif. While WWP1 ubiquitinates RNF11 *in vitro* and *in vivo*, it does not lead to RNF11 degradation or alter its localization. Instead, WWP1 modulates RNF11’s ability to downregulate ErbB2 and EGFR, highlighting its complex role in cancer biology (Chen et al., 2008; Zhi and Chen, 2012).

### 7.2 Substrates without PY motif

WWP1 negatively regulates the TGF-β tumor suppressor pathway by targeting components like Smad2 and TβRI for ubiquitination and degradation, thereby enhancing prostate cancer (PCa) cell proliferation (Massagué and Like, 1985). Notably, TβRI lacks a PY motif, yet WWP1 directly mediates its degradation. Additionally, WWP1 modifies PTEN, another key tumor suppressor that also lacks a PY motif, inhibiting its localization on the plasma membrane and impairing its ability to resist PI3K activation (Lee et al., 2019). This modification increases PI3K signaling, driving uncontrolled growth and drug resistance in tumor cells (Engelman et al., 2006; Song et al., 2012; Fruman et al., 2017; Song and Pandolfi, 2022). WWP1 employs K11 and K48 ubiquitin chains for degradation and adds K27 chains to PTEN, preventing its dimerization without causing degradation, ultimately supporting PCa cell survival.

p27, a key regulator that inhibits the cyclin E/CDK2 complex, also lacks a PY motif but is targeted by WWP1, which facilitates its lysine-48-linked ubiquitination (Cao et al., 2011). In acute myeloid leukemia (AML), elevated WWP1 expression reduces p27 levels, promoting cell cycle progression and enhancing AML cell survival (Sanarico et al., 2018). In liver cancer tissues, KLF14 expression is significantly downregulated, yet it accumulates at the VEPH1 promoter, enhancing VEPH1 transcription and protein expression. However, WWP1 ubiquitinates and degrades KLF14, which lacks a PY motif, suppressing VEPH1 expression and promoting liver cancer cell proliferation, invasion, and migration (Scohy et al., 2000; Zhang et al., 2024).

Additionally, the tumor suppressor protein p53 plays a critical role in cellular stress responses, facilitating growth arrest or apoptosis. WWP1 interacts with the DNA-binding domain of p53, and although the proline-rich domain of p53 lacks a PY motif, it enhances protein-protein interactions. WWP1 ubiquitinates p53 via an E3 ligase-dependent mechanism, promoting its nuclear export and inhibiting apoptosis, thus contributing to tumor progression (Laine and Ronai, 2007; Cheng et al., 2014).

This section explores the regulatory mechanisms controlling upstream modulators and downstream targets, highlighting their roles in proliferation, apoptosis, cell cycle progression, migration, and invasion. These findings underscore the significance of WWP1 in cancer biology (Figure 4).

## 8 WWP1 in cancers

Cancer remains a major global public health concern, ranking as the second leading cause of death after heart disease (Gummlich, 2020; Siegel et al., 2021; Simsone et al., 2021). Current treatment modalities primarily include surgery, chemotherapy, and immunotherapy (Dai et al., 2022; Zhao et al., 2024). However, the limited bioavailability and significant drug resistance associated with anticancer medications present substantial challenges in chemotherapy (Liu et al., 2022; Svensson et al., 2024). This highlights the urgent need for novel tumor-targeting pathways to enhance cancer diagnosis and treatment (Gummlich, 2023). Research has demonstrated that alterations and dysfunctions in E3 ligase genes contribute to the pathogenesis of various diseases, including cancer, with several HECT domain E3 ligases identified as critical regulators in cancer progression and treatment (Bernassola et al., 2019). Given WWP1’s complex role, it is proposed as a potential therapeutic target for various human cancers. This section explores WWP1’s functions across different malignancies, paving the way for innovative cancer therapies (Table 3).

**TABLE 3:** Roles of WWP1 in different tumor types.

Tumor type	Cell line	Expression level in tumor	Signaling pathway	Effects	References
Glioma	U87 U251 LN229	Downregulation	NF-κB	WWP1 promotes cell proliferation through the miR-30a-5p-WWP1-NF-κB positive feedback loop.	Zhao et al. (2019)
Melanoma	CRL-2208 HTB-65 ACC236	Downregulation	PI3K-AKT-mTOR	WWP1 inhibits the proliferation, migration and invasion of melanoma.	Jia et al. (2021)
Hepatocellular carcinoma	Huh7 SNU449 MHCC97H	Higher	p53	WWP1 deficiency inhibits cell growth and induces apoptosis of hepatocellular carcinoma by activating Caspase 3 and p53 expression.	Cheng et al. (2014)
Intrahepatic cholangiocarcinoma	RBE Huh28	Overexpression	N/A	WWP1 promotes the proliferation and invasion of intrahepatic cholangiocarcinoma.	Li et al. (2022)
Papillary Thyroid Cancer	NPA87 KAT-5 B-CPAP	Upregulation	PI3K-AKT	WWP1 promotes cell proliferation and inhibits apoptosis through the PI3K-AKT signaling pathway.	Wang et al. (2022)
Acute myeloid leukemia	CD34	Upregulation	N/A	WWP1 inhibits autophagy signal transduction and increases the survival rate of leukemia cells.	Sanarico et al. (2018)
Breast cancer	MCF10A COS7 MCF7	Overexpression	Hippo	WWP1 promotes the proliferation of breast cancer cells.	Zhao et al. (2012); Zhi et al. (2012)
Osteosarcoma	MG63 HOS	Higher	N/A	WWP1 promotes the invasion of osteosarcoma cells and inhibits apoptosis.	Wu et al. (2015)
Prostatic cancer	PC-3 22Rv1	Upregulation	TGF-β PI3K-AKT	WWP1 promotes the proliferation of prostate cancer cells.	Lee et al. (2019); Chen et al. (2007)
Gastric cancer	MKN-45 AGS	Upregulation	PTEN/AKT	WWP1 promotes cell growth through the PTEN-AKT signaling pathway.	Zhang et al. (2015)
Cutaneous squamous cell carcinoma	A431 SCL-1	Higher	STAT3	WWP1 promotes the growth, migration and invasion of CSCC cells through STAT3 signaling pathway.	Xia et al. (2018)
Colorectal cancer	SW480 HCT116 HT29	Overexpression	PTEN/AKT	WWP1 promotes proliferation and metastasis through PTEN-AKT signaling pathway.	Chen and Zhang (2018)

### 8.1 WWP1 and glioma

**FIGURE 5:**
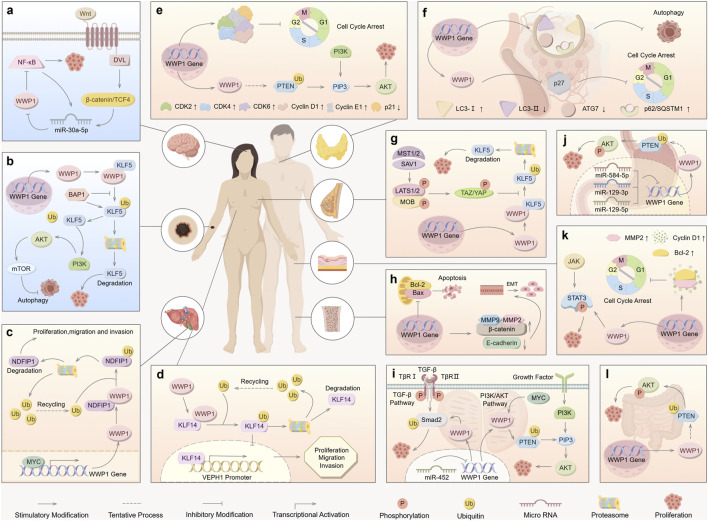
Roles of WWP1 in different types of human cancers. WWP1 functions as both a tumor suppressor (indicated in light blue for glioma and melanoma) and a tumor promoter (indicated in light yellow for other cancer types). **(A)** The Wnt/β-catenin pathway activates miR-30a-5p, which inhibits NF-κB by targeting WWP1, thereby promoting glioma. **(B)** BAP1 inhibits WWP1-mediated ubiquitination of KLF5 in melanoma. **(C)** In ICC, WWP1 ubiquitinates NDFIP1, promoting tumor proliferation, migration, and invasion. **(D)** WWP1 enhances the proliferation, migration, and invasion of HCC cells. **(E)** WWP1 accelerates PTC progression by ubiquitinating PTEN, thereby promoting the PI3K-AKT signaling pathway. **(F)** WWP1 inhibits cell cycle arrest by reducing p27 accumulation, facilitating AML progression. **(G)** TAZ/YAP counteract WWP1-mediated KLF5 ubiquitination, promoting BC cell proliferation. **(H)** WWP1 inhibits apoptosis and induces EMT, promoting OS growth. **(I)** WWP1 facilitates PCa proliferation by activating the TGF-β and PI3K-AKT signaling pathways. **(J)** WWP1 enhances GC cell survival through the PTEN/AKT signaling pathway. **(K)** WWP1 activates the STAT3 signaling pathway and promotes cell cycle progression in CSCC. **(L)** WWP1 supports CRC cell proliferation via the PTEN/AKT signaling pathway.

Glioma, originating from glial or precursor cells, accounts for 80% of malignant tumors in the central nervous system (CNS). Despite surgical resection, radiotherapy, and chemotherapy, patient prognosis remains poor, underscoring the need for novel treatment strategies (Zhang et al., 2021; Wei et al., 2023). Studies suggest that miR-30a-5p promotes glioma cell growth and invasion. Research by Zhao et al. indicated that WWP1 inhibits NF-κB activation, a pathway linked to glioma formation, and is downregulated in glioma tissues, possibly targeted by miR-30a-5p (Wang et al., 2015; Park et al., 2020) (Figure 5A). The Wnt/β-catenin pathway activates miR-30a-5p, which directly binds to β-catenin/TCF4. In glioma cell lines (U87, U251), decreased WWP1 mRNA levels correlate with increased miR-30a-5p and phosphorylated p65 levels. Overexpression of WWP1 reduces NF-κB p65 phosphorylation, while NF-κB p65 overexpression enhances miR-30a-5p via direct binding to its promoter, forming a positive feedback loop in the “miR-30a-5p-WWP1-NF-κB” signaling axis (Zhao et al., 2019).

Although these findings suggest a potential role for WWP1 in glioma development and indicate its promise as a therapeutic target, further direct experimental evidence is required to confirm its causal involvement in glioma progression. The potential for targeting WWP1 in glioma therapy remains a promising area for future investigation.

### 8.2 WWP1 and melanoma

Melanoma, originating from the malignant proliferation of epidermal melanocytes, constitutes only 1% of skin tumors (Cronin et al., 2018), emphasizing the importance of early detection and treatment to reduce mortality (Neila and Soyer, 2011; Saamarthy et al., 2024). Autophagy has been identified as a mechanism that inhibits precancerous cell proliferation, presenting itself as a potential therapeutic target for melanoma (Gozuacik and Kimchi, 2004; Liu et al., 2013; Jia et al., 2019). Studies on various melanoma cell lines (CRL-2208, HTB-65, ACC236) reveal low WWP1 expression alongside high KLF5 levels, which correlate with poor prognosis. This interaction suppresses autophagy while enhancing melanoma migration and invasion, suggesting an essential role for the WWP1-KLF5-BAP1 axis in melanoma progression (Jia et al., 2021) (Figure 5B).

Although these findings underscore the potential involvement of WWP1 in melanoma, further experimental validation is necessary to establish a causal relationship between WWP1 expression and melanoma progression. These insights highlight promising therapeutic targets for addressing this aggressive form of skin cancer.

### 8.3 WWP1 and liver cancer

Primary liver cancer is histologically classified into hepatocellular carcinoma (HCC) and intrahepatic cholangiocarcinoma (ICC). The development of liver cancer is driven by complex pathological mechanisms, including hepatitis infection, alcohol-induced cirrhosis, and dietary factors (Massarweh and El-Serag, 2017; Salazar and Le, 2021). ICC, a highly aggressive and lethal primary hepatobiliary tumor, exhibits poor prognosis and rising global incidence (Joob and Wiwanitkit, 2017). ICC is now recognized as the second most prevalent liver malignancy following HCC (Huang et al., 2021). Literature indicates that the proto-oncogene MYC enhances WWP1 expression (Ambrozkiewicz et al., 2018). MYC-induced upregulation of WWP1 promotes NDFIP1 ubiquitination, contributing to ICC progression (Li et al., 2022) (Figure 5C). Given its pivotal role in ICC pathogenesis, WWP1 serves as a potential prognostic marker and therapeutic target for ICC.

HCC, one of the most prevalent cancers worldwide, is marked by significantly elevated WWP1 protein levels in tumor tissues compared to adjacent non-cancerous tissues. Cheng et al. demonstrated that silencing WWP1 suppresses HCC cell proliferation and activates Caspase 3 and p53, critical mediators of apoptosis that promote programmed cell death in HCC cells (Laine and Ronai, 2007; Cheng et al., 2014). Additionally, KLF14 accumulates on the VEPH1 promoter, enhancing transcription and protein production. However, WWP1-mediated post-translational modifications suppress VEPH1 expression, potentially driving liver cancer cell proliferation, invasion, and migration (Zhang, Wang et al., 2024) (Figure 5D).

While these findings suggest a role for WWP1 in HCC progression, further experimental validation is needed to confirm its precise mechanisms and therapeutic potential in HCC treatment.

### 8.4 WWP1 and papillary thyroid cancer (PTC)

PTC is the most common endocrine malignancy and a leading cause of death among malignant tumors (Turan and Erkılıç, 2023). Current treatments for PTC primarily include surgery along with various alternative therapies (Garcia-Alvarez et al., 2022). Research has shown that WWP1, recognized as a multifunctional protein in PTC, is highly expressed in PTC tissues and cell lines. In PTC cell lines such as Nthy-ori3-1, NPA87, and KAT-5, WWP1 knockout led to increased PTEN levels, resulting in reduced p-PI3K and p-AKT levels. In B-CPAP and TPC-1 cells treated with si-WWP1, the expression levels of CyclinD1, CyclinE1, CDK2, CDK4, and CDK6 were suppressed, while p21 expression significantly increased. Conversely, WWP1 overexpression yielded the opposite effects, promoting PTC cell proliferation and preventing cell cycle arrest (Wang et al., 2022) (Figure 5E). These findings suggest that WWP1 exerts oncogenic effects in PTC cells, making it a potential therapeutic target for PTC treatment.

### 8.5 WWP1 and AML

AML is a blood cancer characterized by distinct genetic mutations (Xia et al., 2023). Its high heterogeneity contributes to frequent relapses, emphasizing the need for targeted therapies (Lin et al., 2022; Nelles et al., 2023). WWP1’s role in AML is modulated through its protein substrates, including p27, p53, and Smads (Peschiaroli et al., 2010; Cao et al., 2011). Specifically, WWP1 inactivation increases p27 levels, inducing cell cycle arrest in AML cells and impairing the malignant potential of bone marrow cells. Additionally, WWP1 inactivation promotes the conversion of cytoplasmic LC3-I to lipid-bound LC3-II, a key marker of autophagosome membrane formation (Klionsky et al., 2016). In AML cells, LC3-I and p62/SQSTM1 levels increased, while LC3-II and ATG7 levels decreased, leading to autophagy inhibition (Sanarico et al., 2018) (Figure 5F). These findings suggest that WWP1 may be a promising therapeutic target for AML, although further studies are necessary to elucidate its mechanisms and develop effective targeted treatments.

### 8.6 WWP1 and breast cancer (BC)

In the Hippo signaling pathway, MST1/2, assisted by its cofactor SAV1, enhances LATS kinase activity by phosphorylating LATS1/2 and the LATS cofactor MOB. Activated LATS1/2 phosphorylates YAP and TAZ, inhibiting their function by promoting their cytoplasmic translocation or degradation (Kanumuri et al., 2021). Research indicates that TAZ/YAP can counteract WWP1-mediated KLF5 degradation, thereby promoting breast cancer cell proliferation and tumor development (Figure 5G). WWP1-mediated ubiquitination contributes to the degradation of LATS1, regulating BC cell proliferation by reducing LATS1 levels (Yeung et al., 2013).

Additionally, WWP1 acts as an inhibitor of bone metastasis in MDA-MB-231 BC cells. Inhibiting WWP1 substantially decreases CXCL12-induced CXCR4 lysosomal transport and degradation, leading to an increased number of osteolytic lesions and metastatic sites (Benovic and Marchese, 2004; Bohn et al., 2009). These findings suggest that factors influencing WWP1 expression or activity could alter the metastatic potential of BC cells to bone (Nguyen Huu et al., 2008).

### 8.7 WWP1 and osteosarcoma (OS)

OS, a malignant tumor arising from mesenchymal tissue, is the most common primary malignancy of the human skeletal system. It typically presents at advanced stages, exhibits high early metastasis rates, and demonstrates resistance to chemotherapy, resulting in low cure rates (Wang et al., 2024). Wu et al. reported that WWP1 mRNA expression was elevated in 88% of OS tissues compared to adjacent non-cancerous tissues, suggesting an oncogenic role for WWP1 in OS. Moreover, WWP1 overexpression increased MMP2 and MMP9 activity, proteins associated with tumor metastasis and angiogenesis. WWP1 also promotes OS cell invasion by modulating MMP2 and MMP9 activity and inducing epithelial-mesenchymal transition (EMT) (Schmalhofer et al., 2009; Wu et al., 2015) (Figure 5H). These findings highlight the potential role of WWP1 in OS pathogenesis and progression, but further studies are required to confirm its precise role and therapeutic potential in OS treatment.

### 8.8 WWP1 and prostate cancer (PCa)

PCa poses a significant public health challenge in developed nations, ranking as the second most common cancer among men and a leading cause of cancer-related deaths in older males (Albertsen, 2020; Zhou et al., 2024). Research has identified the WWP1 gene on chromosome 8q21, a region frequently amplified in various cancers, including PCa, suggesting that WWP1 may function as an oncogene in PCa. WWP1 overexpression has been observed in up to 60% of PCa xenografts and cell lines, such as the 22Rv1 PCa cell line. Studies revealed that WWP1 facilitates the degradation of key components of the TGF-β signaling pathway, thereby enhancing cell proliferation by inhibiting TGF-β signaling (Chen et al., 2007).

Another study demonstrated that miR-452 expression is significantly reduced in PCa tissues, while WWP1 is overexpressed. miR-452 inhibits PCa cell migration and invasion by downregulating WWP1 expression (Goto et al., 2016) (Figure 5I). These findings suggest that WWP1 may serve as a therapeutic target for PCa detection and treatment. However, further studies are necessary to fully elucidate its mechanisms and confirm its potential as a clinical target for PCa therapy.

### 8.9 WWP1 and gastric cancer (GC)

GC is a highly prevalent malignancy, and despite advancements in treatment, survival rates for patients with advanced GC remain low, particularly among those experiencing metastasis, recurrence, or chemotherapy (Yang et al., 2023). Recent studies have implicated the NEDD4-like protein family in cancer progression through PTEN reduction and AKT signaling promotion in tumor cells (Wang et al., 2007). The PTEN/AKT pathway is crucial in oncogenic signaling across various cancer types (Cheng et al., 2008). WWP1 has been shown to ubiquitinate PTEN, increasing p-AKT levels and promoting GC cell growth and proliferation (Zhang et al., 2015).

Furthermore, dysregulated oncogenic and tumor-suppressor miRNAs play essential roles in processes such as cell proliferation, apoptosis, invasion, angiogenesis, and drug resistance in tumors (Song and Ajani, 2013). Using bioinformatics tools such as TargetScan and miRCode, miR-129-5p and miR-129-3p were identified as potential WWP1 regulators (Lewis et al., 2003; Jeggari et al., 2012). Research indicates that these miRNAs suppress WWP1, thereby inhibiting GC cell survival (Ma et al., 2019). Another study revealed a negative correlation between miR-584-5p levels and WWP1 expression in GC tissues and cell lines, with miR-584-5p suppressing GC cell proliferation by downregulating WWP1 (Li et al., 2017) (Figure 5J). These findings highlight WWP1 as a promising target for GC treatment and prognosis, offering valuable insights for novel therapeutic development.

### 8.10 WWP1 and cutaneous squamous cell carcinoma (CSCC)

CSCC is a growing global health concern, with increasing incidence rates and a high propensity for invasion, metastasis, and recurrence (Kivisaari and Kähäri, 2013; Wang et al., 2021). Xi et al. reported that WWP1 mRNA and protein levels were significantly elevated in SCL-1 and A431 cells compared to normal tissues. WWP1 depletion resulted in reduced levels of MMP-2, cyclin D1, and Bcl-2, leading to G0/G1 phase cell cycle arrest. JAK is identified as an upstream kinase of STAT3, and WWP1 enhances CSCC cell proliferation by modulating total STAT3 and p-STAT3 levels, thereby activating the STAT3 signaling pathway (Xia et al., 2018; Xu et al., 2022) (Figure 5K).

These findings suggest that WWP1 may serve as a potential molecular target for CSCC treatment. However, further investigation is needed to fully understand the mechanisms through which WWP1 exerts its influence and to validate these preliminary results. These insights could pave the way for therapeutic strategies, but additional studies are required to confirm WWP1’s role as a reliable molecular target for CSCC diagnosis, prognosis, and treatment.

### 8.11 WWP1 and CRC

CRC is one of the leading causes of cancer-related mortality globally (Aguiar Junior et al., 2020). Despite advancements in screening and preventive measures, CRC remains a significant health challenge due to its high incidence (Villéger et al., 2018). Research demonstrates that transfecting an overexpression plasmid of WWP1 into CRC SW480 cells induces a dose-dependent increase in WWP1 protein levels. Studies suggest that NEDD4, a specific E3 ligase for PTEN, reduces PTEN levels through polyubiquitination (Wu et al., 2003; Trotman et al., 2007), thereby activating the AKT signaling pathway (Trotman et al., 2007). Similarly, WWP1 elevates p-AKT levels by ubiquitinating PTEN in CRC, promoting CRC cell proliferation and metastasis (Chen and Zhang, 2018) (Figure 5L). These findings highlight the significant role of WWP1 in CRC, suggesting its potential as a novel target for CRC diagnosis and treatment. This research lays the foundation for developing future therapeutic strategies centered on protein ubiquitination and antitumor activity stimulation.

## 9 Conclusion and perspectives

E3 ligases play a critical role in tumorigenesis, and targeting these enzymes for drug development has emerged as a challenging yet promising research direction. WWP1, as an E3 ligase, is implicated in various tumorigenic processes, including tumor growth, progression, and metastasis. It significantly influences pathophysiological processes such as the tumor immune microenvironment, cell autophagy, cell cycle regulation, and tumor metabolism. However, its precise mechanisms remain to be fully elucidated (Behera and Reddy, 2023). WWP1 maintains cellular equilibrium by regulating downstream protein expression, influencing tumor progression (Jiang et al., 2020).

WWP1 modulates the expression of several downstream proteins, including TβRI, Smad2, ErbB4/HER4, KLF5, p63, and p27. It frequently targets proteins containing the PY motif, such as Smad2, KLF5, and p63, for ubiquitin-dependent degradation. Additionally, WWP1 regulates substrates lacking PY motifs, such as p53, KLF14, and p27, through adaptor proteins. Despite these insights, the underlying regulatory mechanisms require further investigation (Hu et al., 2004; Ingham et al., 2004). This review summarizes the upstream regulatory factors influencing WWP1 and its associated substrate proteins. Gaining a deeper understanding of these mechanisms could enable the development of effective strategies to inhibit cancer progression by targeting the WWP1 pathway.

By outlining the known mechanisms and roles of WWP1, this review provides a solid theoretical foundation for considering WWP1 as a promising therapeutic target in human cancers.

For example, WWP1 expression is downregulated in glioma tissues, suggesting a tumor-suppressive function (Zhao et al., 2019). Conversely, in cancers such as breast cancer (Zhi et al., 2012) and prostate cancer (Goto et al., 2016), WWP1 functions as an oncoprotein. This duality highlights the complexity of WWP1’s role in tumorigenesis and the necessity of a nuanced understanding of its involvement in different cancer types.

Future research on WWP1 as a potential therapeutic target in cancer should prioritize several key areas. First, a deeper understanding of its role across various cancer types is essential, particularly through patient-derived models that examine its involvement in tumor growth, metastasis, and treatment resistance (Faber et al., 1998). Second, further investigation into the molecular mechanisms by which WWP1 interacts with critical signaling pathways, such as PI3K/Akt and NF-κB, will provide insights into its functional relevance in cancer biology. Additionally, developing specific inhibitors or therapeutic agents targeting WWP1—alone or in combination with chemotherapy or immunotherapy—could improve treatment outcomes. Moreover, exploring WWP1 as a clinical biomarker for cancer diagnosis, prognosis, and therapeutic response will be vital for personalized treatment strategies. Finally, understanding its role in immune evasion within the tumor microenvironment may open new avenues for immunotherapy-based approaches. These research directions will be pivotal for advancing WWP1-targeted therapies and improving cancer treatment outcomes.

In conclusion, WWP1 is a multifunctional protein with complex roles in tumorigenesis. This review systematically highlights WWP1’s potential as both an oncoprotein and a tumor suppressor, offering guidance for developing effective strategies to target WWP1 and impede tumor progression. Furthermore, this review provides a preliminary profile of WWP1’s current status in human cancer research, underscoring its significance as a multifunctional protein. Further studies are needed to explore WWP1’s involvement in diseases beyond cancer. Nevertheless, the distribution and functions of WWP1 reinforce its promise as a therapeutic target for human cancers. Additional research is essential to fully elucidate the mechanisms underlying WWP1’s functions and to evaluate its therapeutic potential across a broader spectrum of human diseases.
